# Recurrent Discoloration After Internal Bleaching Postregenerative Endodontics

**DOI:** 10.1155/crid/8492527

**Published:** 2026-01-16

**Authors:** S. Drouri, M. Batty, S. El Baz, H. El Merini

**Affiliations:** ^1^ Department of Conservative Dentistry and Endodontics, Faculty of Dentistry, University Hassan II, Casablanca, Morocco, uh2c.ac.ma

**Keywords:** bleaching, discoloration, regenerative endodontics, revascularization

## Abstract

**Introduction:**

Regenerative endodontic therapy (RET) has become a preferred treatment for immature necrotic teeth. However, one of the undesirable outcomes is tooth discoloration, which can significantly affect esthetics. Internal bleaching is commonly used, but success rates can vary due to several factors, including the type of materials used during the procedure.

**Case Report:**

We report a case of a 15‐year‐old female patient with severe tooth discoloration following RET on a maxillary central incisor. Initial internal bleaching provided satisfactory results, but the discoloration recurred within a few months. Additionally, the tooth developed an abscess, complicating the clinical management. Endodontic retreatment was performed, but apical access was challenging. Despite successful management of the infection, the tooth discoloration persisted. The persistence of discoloration was attributed to the materials used during the RET, specifically MTA, which has been known to cause color changes over time.

**Conclusions:**

This case highlights the challenges of managing tooth discoloration post‐RET and the limitations of internal bleaching as a treatment. It underscores the importance of considering preventive strategies during the RET procedure to minimize discoloration. Alternative esthetic approaches may be necessary when bleaching fails to meet the patient′s expectations.

## 1. Introduction

Regenerative endodontic therapy (RET) is a revolutionary alternative treatment that allows, unlike traditional root canal therapy, the regeneration of the dentin–pulp complex in a bacteria‐free root and in the presence of the right biological conditions (stem cells, scaffolds, and growth factors) [1]. However promising RET is, this technique still presents many challenges and complications, and tooth discoloration is one of the main concerns [2].

Tooth discoloration, considered a failure based on the patient‐centered outcome [3], is not included in RET failure article cases and therefore not published enough as true failure but considered healing or healed cases.

Tooth discoloration is a frequent occurrence and concern of many patients, caused by many factors including RETs. And many patients perceive this change of color as unesthetic especially when it affects anterior tooth; which may negatively the quality of life and the self‐esteem of many patients [4].

Tooth whitening is often the most cost‐effective, minimally invasive approach to treat discoloration. However, it can be a challenge and a not fully predictable procedure if performed after RETs considering the tooth condition post‐RETs [5].

Thus, the aim of this case report was to present the recurrent discoloration after internal bleaching post‐RETs to understand the causes and how it can be avoided.

## 2. Case Presentation

A 15‐year‐old female was referred to a resident in the conservative dentistry and endodontics department of a hospital affiliated to the Faculty of Dentistry, University Hassan II, Casablanca‐Morocco, complaining of discoloration of Tooth 21. The young patient was accompanied by her mother. The patient′s medical antecedents showed nothing in particular. The patient was apparently healthy.

The patient received a regenerative endodontic procedure in her previously necrotic Tooth 21 in the pediatric dentistry department of the same hospital 7 years earlier.

Clinical examination revealed gray discoloration in the cervical region, making her smile unsightly (Figure 1a,b).

Figure 1(a, b) Anterior view showing discoloration of Tooth 21 (7 years following RET).

**Figure:**
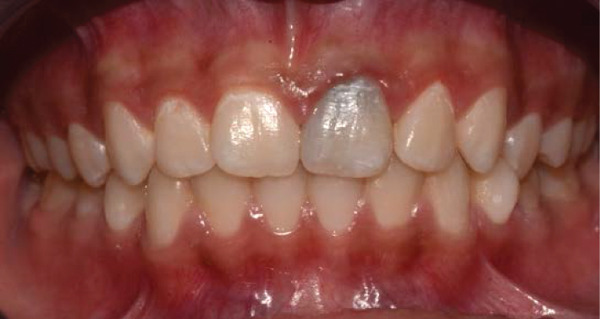
(a)

**Figure:**
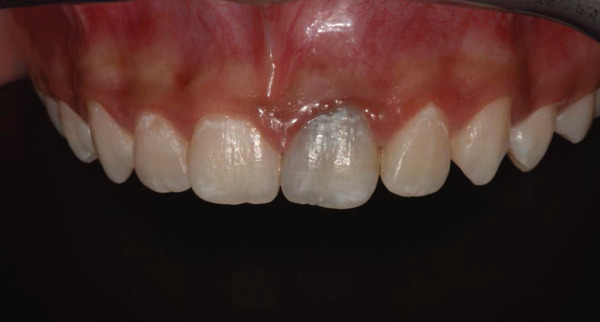
(b)

All procedures for the bleaching treatment and the prognosis for its success were thoroughly explained to the patient and her parent. A written informed consent was signed by the patient for inclusion and consideration in our current study.

**Figure 2:**
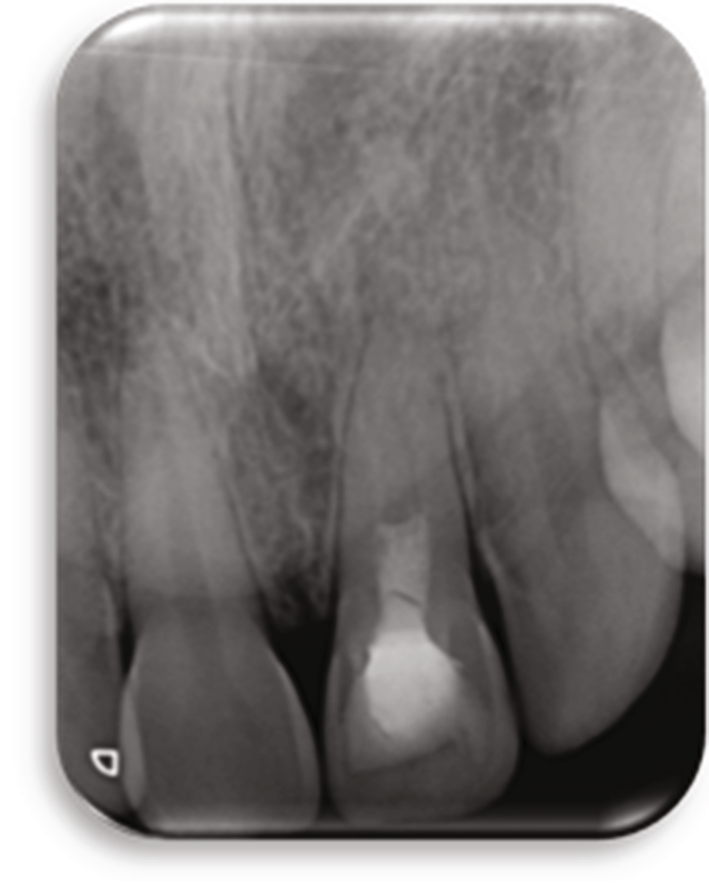
Preoperative radiograph: 7 years post‐RET radiograph showing the placement of MTA and considerable thickening of the dentinal walls and apical closure.

The preoperative periapical radiograph (7 years post‐RET) shows the presence of a well‐adapted cervical mineral trioxide aggregate (MTA) plug and marked thickening of the dentinal walls, with an apparent apical closure suggestive of favorable healing (Figure 2). However, a periapical radiograph alone cannot confirm whether this closure represents a complete and functional apical barrier.

The CBCT images provide crucial additional diagnostic clarity: The canal morphology in the middle and apical thirds is characterized by irregular contours and attenuated dentinal walls. Critically, the CBCT revealed a radiopaque area at the apex not strictly a uniformly mineralized apical closure, but potentially representing a dense tissue bridge. This raises uncertainty about whether a true apical closure was achieved or if persistent apical communication remains a key concern in infection risk (Figure 3).

Figure 3Cone‐beam computed tomography of the case: (a, d) the filling of the coronal part of the root canal with MTA; (b, e) the middle part of the root, the canal appears irregular; and (c, f) axial and coronal views showing a radiopacity attached to the apex.

**Figure:**
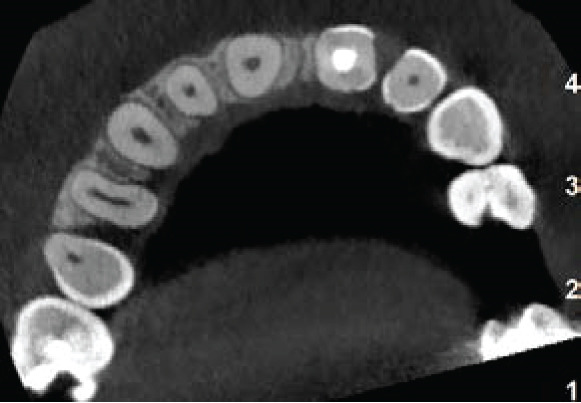
(a)

**Figure:**
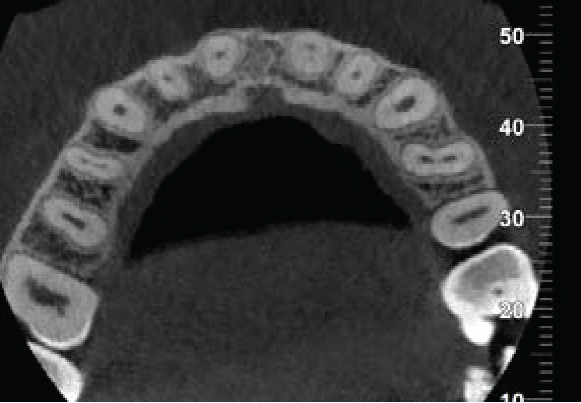
(b)

**Figure:**
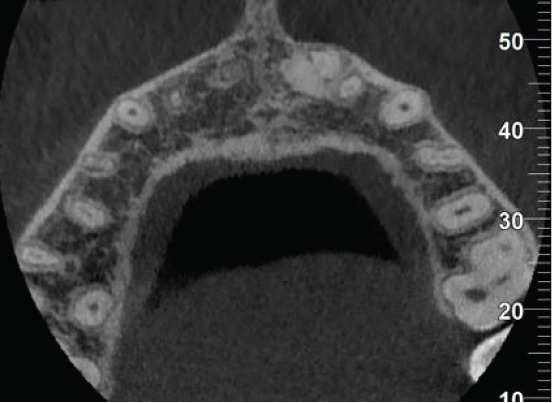
(c)

**Figure:**
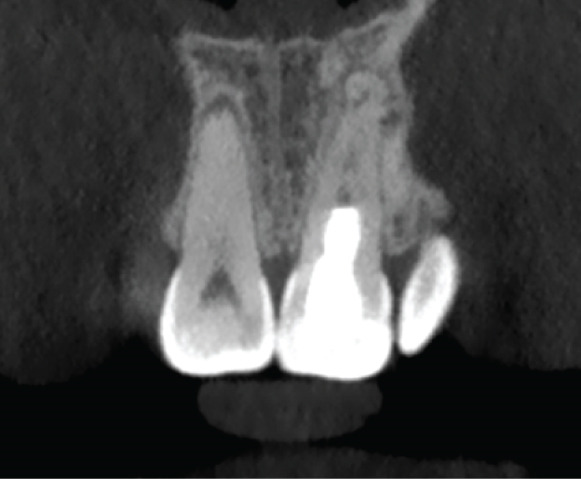
(d)

**Figure:**
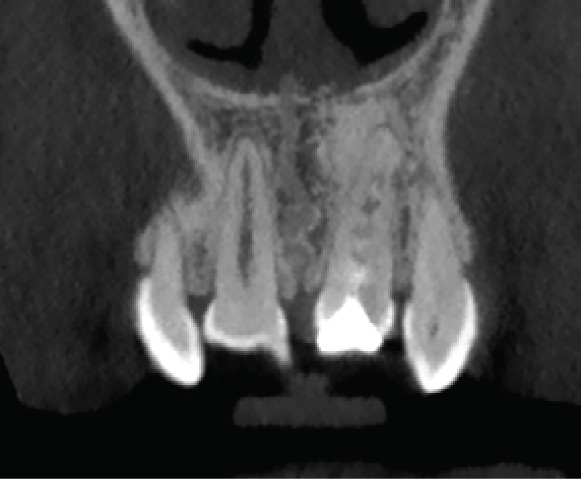
(e)

**Figure:**
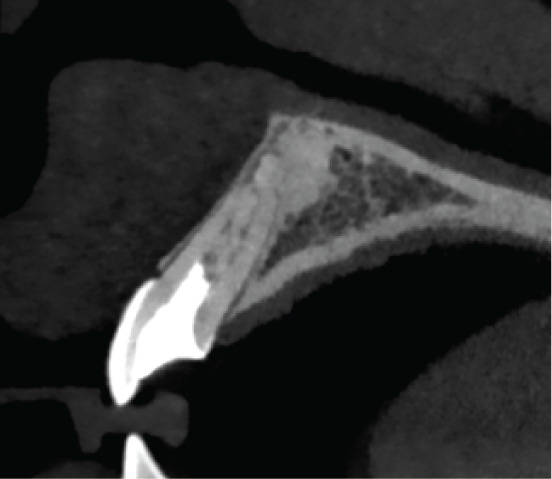
(f)

The prognosis for Tooth 21 is reserved regarding periodontal, endodontic, and esthetic outcomes. We are performing follow‐ups every 6 months to monitor the maturation of the periodontal tissues and will continue this until the patient is ready for a definitive therapeutic solution.

### 2.1. Therapeutic Interventions

**Figure 4:**
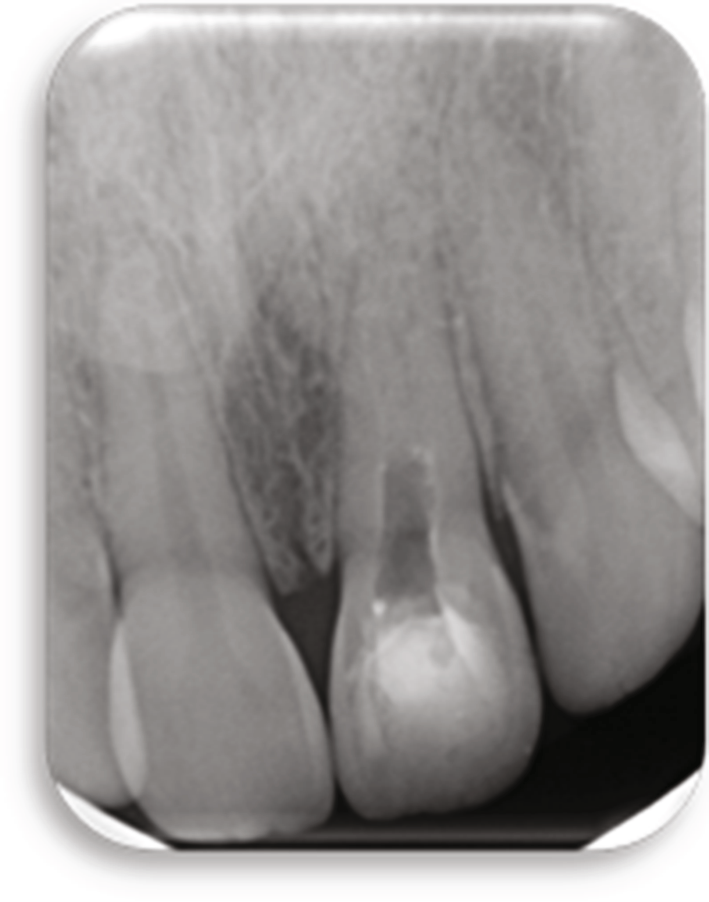
The Pro‐rootMTA was removed; only 1 mm of MTA is left and the glass ionomer was placed.

**Figure 5:**
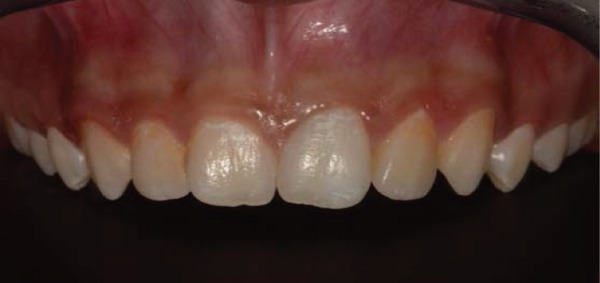
Anterior view of Tooth 21 following 3 weeks with internal bleaching with 37% carbamide peroxide gel.

The Pro‐rootMTA was removed, only 1 mm of MTA is left and a layer of glass–ionomer cement (GIC) (FUJI IX, GC) was placed to create a cervical seal (Figure 4). Internal bleaching was performed three times in 6‐day intervals using 37% carbamide peroxide gel (Figure 5).

**Figure 6:**
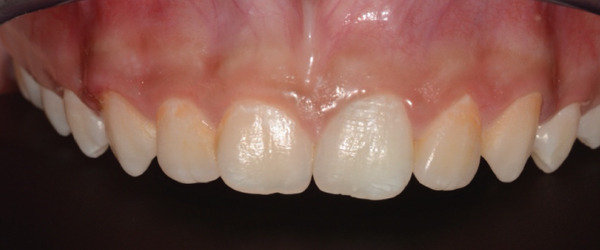
Anterior view of Tooth 21 following 2 weeks of internal/external bleaching showing significant improvement in shade.

At the last review of internal bleaching, the patient and their parent were shown how to apply the bleaching agent inside and outside the dental crown using 10% carbamide peroxide (Opalescence, Ultradent) for 4 h per day over 3 weeks. The result showed a significant improvement in shade (Figure 6). The inside–outside open technique was specifically chosen because of the patient′s young age, allowing the use of lower concentration bleaching agents and thereby reducing the risk of dentinal damage, cervical resorption, or irritation to periapical tissues.

During the last session, the bleaching agent was removed from the access cavity, and the cervical area was thoroughly cleaned and then restored with glass ionomer (FUJI IX, GC). The access cavity was definitively restored with composite material after 2 weeks.

### 2.2. Follow‐Up and Outcome of Interventions

**Figure 7:**
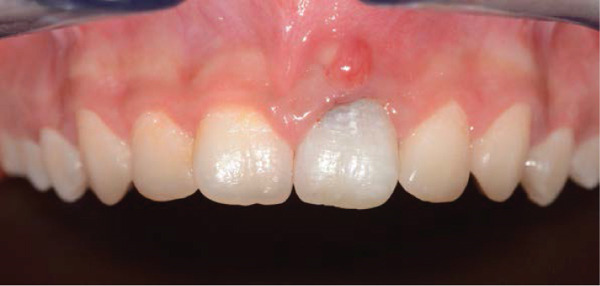
Anterior view showing an acute apical abscess on tooth.

**Figure 8:**
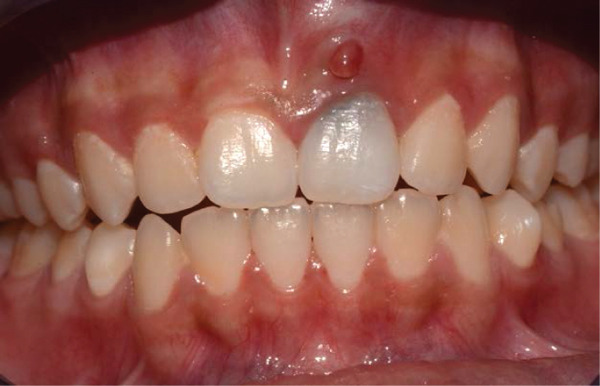
Anterior view of Tooth 21 after 1 week.

Then, 3 months follow‐up, the patient consulted for painful symptoms due to a periapical infection of Tooth 21 (Figure 7). We prescribed antibiotics and pain medications and reviewed the patient 1 week later for the endodontic treatment of Tooth 21 (Figure 8). Anesthesia was administered, the tooth was then isolated, and access cavity was performed. Endodontic treatment was attempted, and the canal was carefully negotiated. Access to the apical region proved to be quite complex due to alterations in canal morphology following revascularization. An appropriate material was used to minimize the risk of damage to the regenerated apical tissue, as evidenced by the radiograph showing the fine Schilder plugger within a root canal (Figure 9a), which demonstrates the maximum length achieved after ultrasonic activation at the cervical third. The canal was well disinfected with 5% sodium hypochlorite (NaOCl) and activated at low frequency using ultrasonic activation (Irrisafe n20–21 mm) to avoid affecting the newly formed tissue. The canal was dried, and calcium hydroxide was placed for intracanal medication (Figure 9b).

Figure 9(a) Assessment with a fine gauge of the limit reached after ultrasound‐assisted access to the coronal third. (b) Placement of a calcium hydroxide filling at the apical limit reached. (c) Radiograph showing the gutta‐percha cone correctly positioned in the canal, with the cone reaching the maximum accessible working length. (d) Postoperative radiograph following endodontic obturation with a single‐cone technique and BioRoot sealer.

**Figure:**
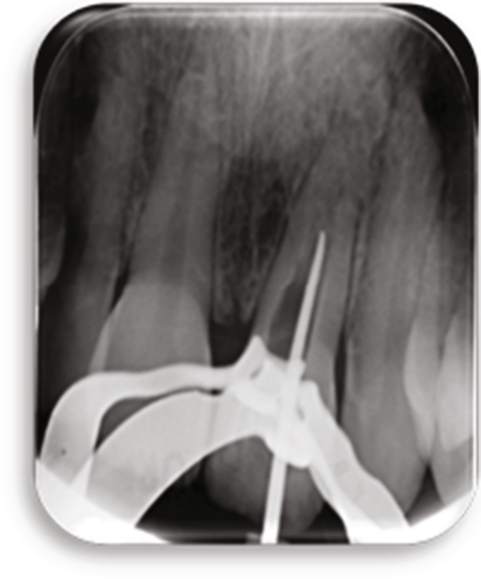
(a)

**Figure:**
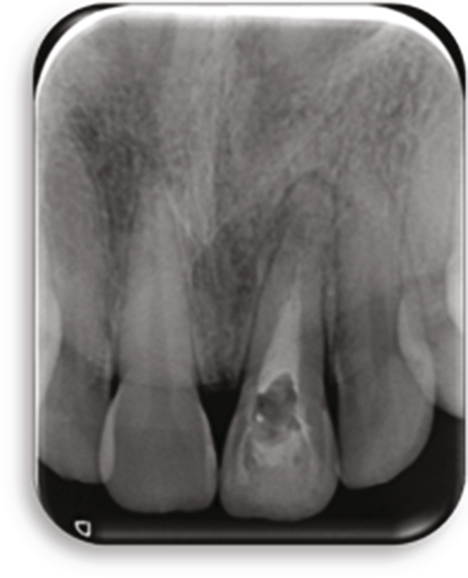
(b)

**Figure:**
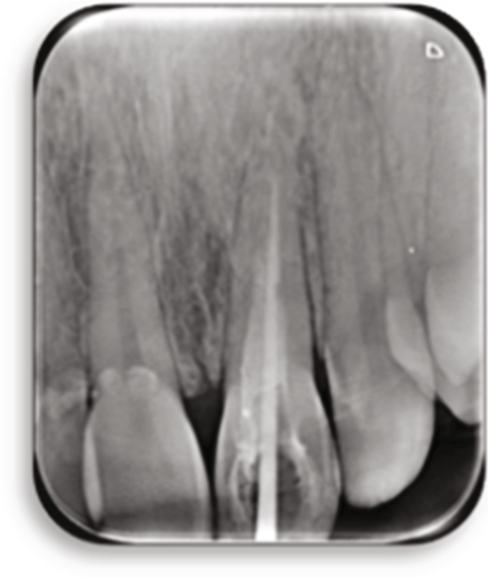
(c)

**Figure:**
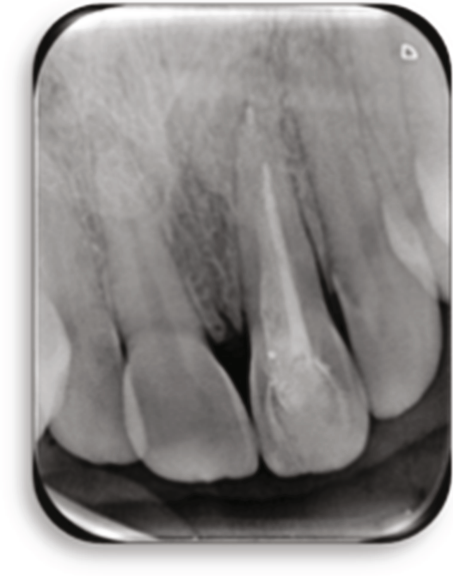
(d)

At the next visit, the clinical signs and symptoms were evaluated. Signs and symptoms of infection had disappeared, and the disinfection procedure was repeated as performed during the last visit. The radiograph showing the cone in place (Figure 9c) demonstrates the apical limit achieved following preparation of the apical third. The calcium hydroxide was renewed for another week. The canal was obturated to this working length using a single‐cone technique and BioRoot sealer (Figure 9d).

**Figure 10:**
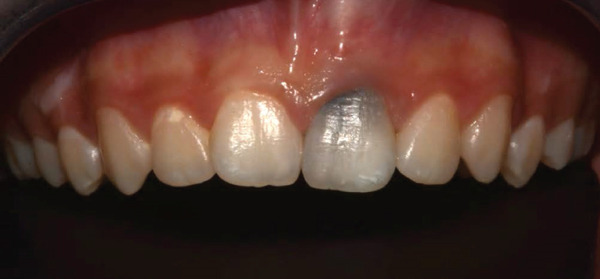
Anterior view of Tooth 21 1‐year follow‐up appointment.

Recurrence of discoloration in Tooth 21 at 1 year is shown in (Figure 10), which could be due to staining of residual MTA fragments or stained MTA components migrating coronally, and rebleeding deposition from periapical inflammation that may have discolored the coronal dentin.

The patient expressed satisfaction with the preservation of the tooth and the comprehensive follow‐up care. While concerned about the recurrence of discoloration and the need for additional interventions, the patient appreciated the clear communication regarding treatment options and reported a positive overall experience with the management process.

## 3. Discussion

### 3.1. Discoloration After RETs

Discoloration after RETs is often due to a combination of the materials used, the breakdown of blood products, and the interaction between these elements within the tooth. Understanding these causes is crucial for clinicians to anticipate, prevent, and manage tooth discoloration.

#### 3.1.1. Intracanal Medicaments

Triple antibiotic paste (TAP) used in the disinfection phase can cause the discoloration. Minocycline, in particular, binds to calcium ions in the dentin and forms insoluble complexes that darken the tooth [6].

The American Association of Endodontists recommends the use of calcium hydroxide as a disinfectant paste to limit discoloration problems [7]. However, concerns have been raised that direct contact with this highly alkaline medicament may inhibit the potential for increasing root canal wall thickness on the dentin surface [8].

#### 3.1.2. Cervical Seal

MTA is commonly used in RETs for its excellent sealing properties and biocompatibility. However, the interaction of bismuth oxide with collagen present in tooth tissue and with remaining NaOCl from canal irrigation can result in the transformation of yellow bismuth into a black precipitate [9].

It is essential for clinicians conducting RET to recognize the significant risk of tooth discoloration during the procedure and follow and take into account certain recommendations [7, 10]:

• A detailed written informed consent should be obtained prior to treating a tooth with RET. It is important to discuss the risks of tooth discoloration and the need for future bleaching/restorative treatment.
• The coronal portion of the tooth should be cleaned of any necrotic pulpal tissues.

#### 3.1.3. Irrigation and Canal Preparation

• Use 20 mL of 1.5%–3% NaOCl for gentle irrigation, ensuring minimal extrusion into the periapical space by employing a needle with a closed end and side vents, or an EndoVac system.
• Follow with saline or EDTA irrigation to minimize cytotoxicity and position the needle 1 mm from the root end.
• Dry canals using absorbent paper points.

#### 3.1.4. Medicaments

• Place calcium hydroxide or a low‐concentration TAP. If TAP is used, seal the pulp chamber with a dentin bonding agent to reduce the risk of staining.
• Deliver the medicament via syringe into the canal system, keeping it below the cementoenamel junction (CEJ) to avoid crown staining.
• The use of minocycline‐containing antibiotic mixtures should be avoided.

#### 3.1.5. Inducing Bleeding

• Create bleeding in the canal by over‐instrumenting 2 mm beyond the apical foramen, filling the canal with blood up to the cemento‐enamel junction. Alternatively, use platelet‐rich plasma (PRP), platelet‐rich fibrin (PRF), or an autologous fibrin matrix (AFM) instead of blood clot formation.

#### 3.1.6. Coronal Barrier

• Place a resorbable matrix (e.g., CollaPlug, Collacote, and CollaTape) over the blood clot if needed, followed by white MTA as the capping material.
• Since MTA can cause discoloration, consider alternatives like bioceramics or tricalcium silicate cements (e.g., Biodentine and EndoSequence BC RRM‐Fast Set Putty) for esthetic cases.
• Apply a 3–4‐mm layer of glass ionomer (e.g., Fuji IX) over the capping material and light‐cure for 40 s.

### 3.2. Factors Influencing Whitening Efficiency After RETs

Tooth whitening for discolored teeth following RETs is the most reasonable first choice of treatment, as it is effective, simple, and minimally invasive [2]. The results are satisfactory for the majority of clinical cases [1]. However, in some cases of discoloration after treatment with TAP, the whitening gave the opposite of the desired effect; the teeth became redder, darker, and less yellow (the value of *L* and *b* decreased whereas the value of a increased) [5].

#### 3.2.1. Whitening Agents/Techniques

Sodium perborate mixed with water is reported to be the safest bleaching agent [11]. However, its effectiveness on severe discoloration is limited. Hydrogen peroxide (H_₂_O_₂_) is more potent and may be more appropriate for cases of intense discoloration [9].

Comparisons of whitening effects on teeth discolored by different antibiotic combinations have shown that 35% H_₂_O_₂_ is more effective than sodium perborate [12].

Then, 10% carbamide peroxide used in external home bleaching has shown efficacy in whitening discoloration caused by MTA [13].

Internal bleaching (walking bleach technique) results in clinically noticeable color changes, though it may not fully restore the tooth′s original shade. Thermo/photo‐activated bleaching may be more effective, but it carries a higher risk of root resorption [14].

Nd‐YAG laser irradiation on a 35% H_₂_O_₂_ increased the efficacy of internal bleaching [2].

#### 3.2.2. Timing of Whitening

Delayed application of bleaching agents (up to 6 months posttreatment) has been shown to mitigate adverse effects on surface microhardness in biodentine; however, postponement does not appear to benefit teeth treated with MTA [9].

#### 3.2.3. Cervical Seal

In many cases of tooth discoloration following RET, a cervical barrier of MTA or other hydraulic calcium silicate–based cements has already been applied. Some authors therefore recommend placing a resin‐modified glass ionomer over the MTA prior to the bleaching process [15]. Others suggest removing 1 mm of MTA and placing the resin‐modified glass ionomer [16]. Complete removal of MTA might independently improve color, as most of the discoloration is within the MTA and not the dentin [17]; however, this would increase the risk of disrupting the seal [9].

### 3.3. Management of the Discoloration

Management of tooth discoloration in non‐vital teeth, especially after RET, can be achieved through various whitening techniques that are effective yet conservative in preserving tooth structure. The walking bleaching technique is a common choice, and its protocol begins with creating a tissue‐conserving access cavity to expose and thoroughly clean the pulpal cavity. The root canal filling material is reduced by 2–3 mm below the gumline, with the position verified by a periodontal probe. An impermeable base, preferably resin‐modified glass–ionomer cement (RMGIC), is placed over the root canal filling to ensure a good apical seal [18].

The pulpal cavity is cleared of any remaining root canal filling, and a bleaching gel is introduced. The access cavity is then temporarily sealed with provisional materials, where RMGIC is recommended for its superior sealing properties compared to conventional GIC. Among provisional restorative materials, Cavit and Coltosol have shown greater effectiveness in maintaining the bleaching agent within the cavity, outperforming options like zinc oxide eugenol and Fermit [18].

On follow‐up, a few days after the initial application, the bleaching result is evaluated, and if necessary, the bleaching agent is reapplied. The timing for reapplication can be adjusted based on the dentin′s age and structural characteristics, as young and old dentin display different rates of hydrogen diffusion, which influences bleaching outcomes [18].

Various bleaching agents can be employed in the walking bleaching technique, tailored to the severity of the discoloration. Sodium perborate mixed with distilled water is a common choice for internal bleaching, given its efficacy and safety profile. In regions where sodium perborate is restricted, 18% carbamide peroxide can serve as a viable alternative. For cases of more intense discoloration, 30%–35% H_₂_O_₂_ or a mixture of sodium perborate with 3% H_₂_O_₂_ is often recommended to achieve optimal results [19].

#### 3.3.1. Inside–Outside Open Technique

The inside‐outside open technique presents a simultaneous bleaching approach by applying the bleaching agent both inside the pulp chamber and externally. Here, the access cavity is kept open, and the root filling is protected by a base. The patient then self‐applies 10% carbamide peroxide in the cavity and an external tray every 4–6 h. After 2–3 days, they return for evaluation of the bleaching progress [19].

#### 3.3.2. Inside–Outside Closed Technique

This technique combines the traditional walking bleach with an external tray specifically for a single tooth, expediting the bleaching process and reducing required visits [19].

#### 3.3.3. Lasers

Laser‐assisted bleaching is another option; Nd laser irradiation has been shown to enhance the bleaching effect of sodium perborate. Despite this, recent findings indicate that while laser activation accelerates the process, final results do not significantly differ compared to bleaching without lasers, irrespective of laser power settings [19].

#### 3.3.4. Cold Atmospheric Plasma (CAP)

CAP has emerged as a novel approach, where plasma is generated through dielectric barrier discharge, utilizing the glass electrode as the primary electrode and the tooth as the secondary electrode. This technique uniquely does not require root canal filling, a protective barrier, or bleaching agents, distinguishing it from traditional methods. However, CAP necessitates specialized equipment due to its advanced mechanism [19, 20].

When the whitening procedures are unsuccessful, ceramic restorations could be the treatment of choice [21]. Patients should be informed of the greater tooth structure loss, higher costs, and risks such as repairs or replacements over time of the prosthodontic alternatives [19].

To the best of our knowledge, this is the first published case reporting a recurrence of discoloration following bleaching of a previously revascularized tooth. This recurrence may be related either to residual bismuth oxide within the dentinal walls—known for its long‐term discoloration potential—or to an insufficient thickness of the glass ionomer barrier over the MTA plug, which may have permitted chemical interactions between the bleaching agents and the underlying materials.

Several limitations must be acknowledged. The risks of dental tissue alterations and unpredictable diffusion of bleaching agents are heightened by the variability in tissue maturation and the fragility of dental structures after RET. Moreover, teeth treated with such procedures have been reported to be more prone to discoloration and reduced fracture resistance. Therefore, a thorough three‐dimensional assessment and a case‐specific risk–benefit analysis are essential to ensure the safety and effectiveness of bleaching.

Dental bleaching should be approached with caution and should not be considered a routine esthetic procedure, particularly in teeth exhibiting variable healing responses and structural changes following regenerative therapy.

## 4. Conclusion

Tooth discoloration is a frequent side effect of regenerative endodontic treatment. Patients need to be appropriately informed about the risk of discoloration and its management, while clinicians should be aware of the procedural aspects that may reduce this risk and the options available for managing it.

## Consent

The patient is fully aware of the implications of publications and accepts any associated risks. The patient has signed the consent.

## Conflicts of Interest

The authors declare no conflicts of interest.

## Author Contributions

S. Drouri was responsible for writing the case presentation, editing the whole manuscript, in addition to proofreading. M. Batty was responsible for writing the introduction and discussion. S. El Baz was responsible for clinical procedures. H. El Merini was responsible for supervision and review. All authors contributed to the drafting of the manuscript and critically revised and edited the manuscript before approving the final draft.

## Funding

This research received no external funding.

## Data Availability

The data that support the findings of this study are available from the corresponding author upon reasonable request.
